# Why People Conceal Mental Health Problems: Qualitative Analysis of TikTok Posts

**DOI:** 10.2196/88244

**Published:** 2026-05-19

**Authors:** Chloe Roske, Kael Ragnini, Qinchun Zhu, Ashari Palmer, Meredith R Kells, Heather A Davis, Matthew K Nock

**Affiliations:** 1 Department of Psychology, Harvard University, 33 Kirkland Ave, Cambridge, MA, 02138, United States, 1 6464775894; 2 School of Nursing, University of Rochester, Rochester, NY, United States; 3 Department of Psychology, Virginia Tech, Blacksburg, VA, United States; 4 Department of Psychiatry, Massachusetts General Hospital, Boston, MA, United States; 5 Department of Psychiatry, Boston Children’s Hospital, Boston, MA, United States

**Keywords:** concealment, disclosure, suicide, social media, TikTok

## Abstract

**Background:**

Concealment of psychiatric symptoms is a barrier to effective mental health treatment, particularly among patients with suicidal thoughts and behaviors. Prior research on concealment has relied on retrospective self-report or laboratory-based interviews, which may not capture real-world decision-making about disclosure. Social media platforms such as TikTok provide a context in which individuals publicly narrate their experiences about concealing psychiatric symptoms, offering insight into motivations for concealment uninfluenced by experimenter demand characteristics.

**Objective:**

To understand patient decision-making about when to conceal and when to disclose psychiatric symptoms, this study examined social media content about patient experiences of concealing mental health symptoms. TikTok was chosen because it is the fastest-growing social media platform, and social media platforms provide an open-ended format for people to express their thoughts and feelings on various topics.

**Methods:**

Using a newly created TikTok account to minimize algorithmic bias, we identified and downloaded the 25 most-viewed English-language videos from 4 search terms about concealment in clinical contexts (“lying to therapist,” “lying to my therapist,” “lying to doctor about mental health,” and “lying to doctors about mental health”). After exclusions, 98 videos were included in the analysis. Videos were analyzed using reflexive thematic analysis. Four coders collaboratively developed a codebook through iterative review, triangulation, and consensus discussions. Engagement metrics (views, likes, comments, shares, saves) were recorded and summarized.

**Results:**

The 98 videos had 73,252,531 views, 14,356,874 likes, 74,954 comments, 770,027 shares, and 1,204,006 saves. Four themes were constructed among the 90 videos that explicitly discussed motivations for concealment: (1) disclosure perceived as punitive (31/90, 34.4% of videos), including desire to avoid hospitalization (17/90, 18.8%); (2) managing others’ feelings and impressions (28/90, 31.1%), including fear of upsetting therapists (5/90, 5.5%) and maintaining a façade of wellness (7/90, 7.7%); (3) negative emotions or inability to identify feelings (21/90, 23.3%), including fear of vulnerability (6/90, 6.6%); and (4) negative opinions of psychiatric treatment (17/90, 18.8%), including concerns about confidentiality (3/90, 3.3%). An exploratory theme captured ambivalence and guilt surrounding nondisclosure.

**Conclusions:**

Results provide insight into patient motivations for concealing their suicidal thoughts and behaviors and offer potential avenues for improving rates of disclosure, which is critical to reducing death by suicide. TikTok creators frequently described concealment as a strategy to avoid perceived punitive consequences, manage interpersonal dynamics, or cope with emotional distress. Findings suggest that current risk management practices and stigma surrounding psychiatric care may unintentionally reinforce concealment behaviors. These insights may inform interventions aimed at improving the therapeutic alliance, enhancing transparency around hospitalization criteria, and reducing barriers to honest reporting of suicide risk.

## Introduction

Approximately 50% of people in the United States are diagnosed with a psychiatric disorder during their lifetime, yet few seek mental health treatment [1,2]. Of those who do, many conceal the extent and existence of symptoms from clinicians, which limits the delivery of adequate treatment [3-5]. Concealment has been associated with worse therapeutic outcomes [6] and may be most consequential for patients with suicidal thoughts and behaviors (STBs) [7]. If a clinician is unaware of a patient’s suicidal thoughts or intent, they may not provide evidence-based treatments for STBs [8] and ultimately be unable to intervene to prevent death by suicide. Indeed, over half of suicide decedents denied suicidal ideation (SI) before their deaths [9,10]. This study sought to better understand when and why patients choose to conceal their psychiatric symptoms from clinicians by conducting a review of social media content.

Around 90% of people conceal significant information from their clinicians, with that information most frequently being about STBs [5]. A study of psychiatric inpatients showed that 51.5% withheld some information about SI during admission [11]. Another study reported that fewer than half of people with SI reported it to a health professional. Of those who did disclose, 25% reported SI to a psychologist, 13% to a psychiatrist, 12% to a counselor, and 2.6% to a social worker [12]. Alarmingly, half of the people who died by suicide denied SI in the month before their deaths [10] and two-thirds denied SI when last asked [9]. Research has shown that patients may choose to conceal or minimize the severity of their STBs for myriad reasons, including shame, embarrassment, the quality of the therapeutic alliance, and feeling misunderstood. People also endorse being afraid of stigmatizing responses from health care professionals, involuntary hospitalization, and being encouraged to take psychiatric medication [4,7,13-18].

Prior work on concealment in clinical contexts may be limited by threats to internal and external validity. To date, research on concealment of STBs has relied on self-report and interview-based methods conducted in research settings. However, people who do not disclose STBs to their treatment team may experience similar deterrents to disclosure in a research context. Thus, studies that included qualitative interviews in a laboratory setting [4,13,16,17] or self-report questionnaires [12,13,19,20] may not wholly measure the reasons why patients conceal information from their clinicians. Indeed, people are prone to engage in self-presentation processes, where people manage the impressions they make on others based on context [21], and demand characteristics are a known limitation in research [22]. As such, existing research may not fully capture reasons for concealment given the research context. More observational work is needed to deepen our insight into why patients choose to conceal or minimize their mental health symptoms. A better understanding of this phenomenon may help improve the identification of people at risk for suicide and other related mental health problems. To our knowledge, no prior study has examined motivations for concealment of psychiatric symptoms using real-world, unsolicited disclosure outside of clinical or laboratory settings.

Social media may offer more naturalistic insight into reasons why patients conceal information from their treatment teams. People often turn to social media when seeking peer support for psychiatric and nonpsychiatric illnesses and engaging in discourse about mental health [23-27]. TikTok, a platform for sharing short-form videos, has recently begun to dominate the social media landscape; it has over 1 billion users per month who spend an average of 10 hours per week on the app [28]. As a result, TikTok hosts a vast collection of new, user-generated content on subjects ranging from eating disorder treatment to discussion of mental health symptoms. This content is unsolicited and voluntarily provided outside the presence of researchers or clinicians. Unlike structured interviews or surveys, these disclosures are not cloaked in institutional authority and may be more ecologically valid. In other words, social media content allows researchers and clinicians to learn what patients discuss outside of the research setting or therapy room. Importantly, mental health-related content posted online can be reliably correlated with the poster’s real-world behavior. For example, predictive language models have been able to identify language used in Facebook posts as a significant predictor of depression diagnoses in corresponding samples’ medical records [29].

TikTok’s specific features and characteristics may lower disclosure barriers. For one, filters, green screen effects, and pseudonymous usernames facilitate some anonymity. Even without these tools, some work suggests that web-based environments can make people feel psychologically distanced from their real-world identities, even if they are not literally anonymous [30]. Perceived anonymity or distance is important because previous work shows that mental health symptom endorsement is higher when surveys are anonymous [31] and social media self-disclosure is higher with anonymity [32]. Research on context-dependent privacy outside of video-based social media further suggests that willingness to disclose sensitive information is shaped not only by identifiability or audience, but also by the perceived tone and seriousness of the environment. Individuals are significantly more likely to disclose stigmatized or illicit behaviors when privacy concerns are cognitively suppressed by playful or informal contextual cues, even when objective privacy risks are constant [33]. TikTok’s entertainment-oriented context may similarly reduce the salience of privacy and consequence, enabling users to share mental health experiences that might feel too risky or inappropriate to relay in research or clinical settings. Importantly, however, disclosure and self-presentation are 2 separate processes. People may disclose but curate how and when they do so.

Indeed, disclosure in online settings is not free from impression management. Certain features of online communication (eg, asynchronicity, visible audience feedback) may, in fact, intensify it [34]. Because TikTok is a video-based platform, motivation to engage in self-presentation is likely activated. However, the value of examining TikTok content does not lie in the assumption that users are completely anonymous or unconcerned with how they are perceived. Rather, it lies in observing how individuals choose to frame and narrate their mental health experiences in a context that differs from clinical encounters. The imagined audience on TikTok is diffuse, peer-oriented, and governed by platform-specific norms rather than institutional authority, diagnostic implications, and material consequences. Consistent with Goffman’s theory of self-presentation, TikTok disclosures can be understood as curated performances oriented toward this imagined audience [35,36]. As a result, while impression management remains salient, the form it takes likely differs meaningfully from that of clinical disclosure, potentially shaping what individuals choose to disclose, emphasize, or withhold. TikTok, therefore, offers a novel perspective on how individuals frame, explain, and justify decisions to disclose or conceal psychiatric symptoms and, therefore, addresses key limitations in prior interview-based research.

Because suicide risk assessment relies heavily on patient self-report, concealment of suicidal thoughts may directly impede identification and intervention. Therefore, understanding these motivations is critical not only for improving therapeutic alliance but also for strengthening suicide risk detection and prevention efforts. To gain more organic insight into patient motivations for concealing mental health symptoms, this study describes TikTok content about concealing mental health symptoms from clinicians using reflexive thematic analysis. Given previous work [4,16,18], the themes constructed will likely surround psychiatric care, particularly involuntary hospitalization, stigma, and feeling isolated.

## Methods

### Ethical Considerations

The Harvard University Institutional Review Board reviewed this study and determined that it does not constitute human subjects research under US Department of Health and Human Services regulations and US Food and Drug Administration regulations (Protocol #IRB24-1553). This determination was based on social media posts being considered public domain and no interaction with content creators. Accordingly, no informed consent was required. Identifying information about content creators is not included in this manuscript to protect their privacy, and only publicly posted videos are included in this analysis. This approach aligns with previous work on samples of short-form social media videos [37-40]. Videos were assigned numeric identifiers to protect user privacy and treated as unique participants.

### Sampling Strategy

Consistent with previous qualitative analyses of TikTok content [24,37,38], we downloaded 100 of the most viewed videos for analysis with search terms we chose a priori. Unlike previous studies [23,37,41], our topic of interest did not neatly align with viral hashtags and search terms (eg, #mentalhealth, #WhatIEatInADay, #dietpills). We explored potential search terms using a new TikTok account to avoid algorithms that tailor content to individuals’ watch history, which is in line with prior work [24,37,38]. A list of potential search terms was created; terms were added if the first 10 videos that appeared broadly addressed motivations for symptom concealment. We observed that the online discourse about concealment did not seem to unify around a single search term. To mirror this, we chose to use 4 terms that had the most relevant content and chose the 25 most viewed videos from each (the TikTok search feature displays videos in order of popularity). This ensured that we were analyzing videos that had the widest reach and may have resonated with the largest audience. Given that the goal of this study was to understand why patients would not disclose psychiatric symptoms to clinicians, we included the 25 most popular videos from the following search terms: “lying to therapist,” “lying to my therapist,” “lying to doctor about mental health,” and “lying to doctors about mental health.” These search terms were chosen because the videos watched in the exploratory phase most consistently addressed the research question. After finalization of search terms, a new TikTok account was created. On January 11, 2025, author CR downloaded the videos, excluding videos that were not in English or made by a creator who self-identified as a clinician. Two videos were excluded because the creator was a clinician. After exclusion, we had a sample of 98 videos and recorded the number of views, likes, comments, saves, and shares. This was done via TikTok data scraping tool Apify.com as well as manual confirmation. No transcription software was used.

### Data Coding and Thematic Analysis

A reflexive thematic analysis approach, following Braun and Clarke’s framework [42,43], was used to collaboratively and inductively construct codes and themes from the data. Data coding procedures are described in Table 1. This approach included researcher triangulation, a method to resolve discrepancies in coding by checking data created by one team member against the others [44]. The codebook was created using a subset of the data, and once it was finalized, the team viewed the final videos at least 3 times and coded them. Themes and subthemes were generated collaboratively after 2 final viewings of the entire sample and a collective agreement that the generated themes robustly described the sample’s content. Quantitative statistical analyses were conducted in R (R Foundation for Statistical Computing) [45], including descriptive statistics about the views, likes, comments, saves, and shares of the videos and the number of videos with each code. Data analysis procedures followed previous work examining TikTok content [24,37,38]. This study was conducted and reported in accordance with the Standards for Reporting Qualitative Research guidelines [46].

**Table 1.** Procedure for reflexive thematic analysis of TikTok videos.

Phase	Description	Authors
Phase I: familiarization with the data	Three coders viewed all the videos twice.	CR, AP, QZ
Phase II: generation of preliminary codes	The same coders rewatched a randomly selected 15% of the sample (n=15) and generated preliminary codes for those videos. The group met for debriefing, which included researcher triangulation, and reflecting on and resolving discrepancies. Preliminary codes for each video were then agreed on. This process was done again with another 15 randomly selected videos. The first author used the agreed on preliminary codes to generate an initial codebook.	CR, AP, QZ
Phase III: revision of codes	The 3 coders watched the first 50 videos 3 times each and independently coded the videos. The coders met to cross-reference each other’s codes and resolve discrepancies with a consensus. New codes were added if the authors thought a video was not being sufficiently captured by the existing codes.	CR, AP, QZ
Phase IV: coding all videos	The codebook was used to code the remaining videos. The coders met to conduct researcher triangulation and resolve discrepancies, involving an additional coder (KR) for added perspective.	CR, AP, QZ, KR
Phase V: creating themes	The 4 coders rewatched every video in the sample twice, reflected on the codebook, and met to discuss larger patterns in the data. Four overarching themes were constructed with 4‐7 subthemes each.	CR, AP, QZ, KR

This study was guided by a constructivist lens, which assumes that people construct meaning through their experiences and social contexts. Thus, videos were not considered to reflect a single objective reality. We interpreted videos as personal, context-dependent narratives shaped by creators’ identities, audiences, experiences, and social lives. The research team included college undergraduates, doctoral trainees, and faculty with varied levels of clinical and research experience. The team was diverse with respect to race, ethnicity, age, and sexual orientation. Doctoral trainees and faculty acknowledged that their clinical training and prior work in suicide risk assessment could influence the interpretation of concealment narratives. Undergraduate coders were therefore explicitly encouraged to offer their interpretations and to challenge senior team members when discrepancies arose. The inclusion of college-aged coders was particularly valuable given that many content creators appeared to be adolescents or young adults. Their perspectives helped contextualize language, humor, and platform norms. Reflexive discussions were integrated throughout the coding process to critically examine assumptions, address potential bias, and promote analytic rigor.

In Phase V (developing themes), the research team carefully examined videos (n=12) that had been assigned codes about feelings surrounding concealment of psychiatric symptoms. In doing so, they observed that a subset of coded videos (n=8) discussed concealing mental health symptoms but did not elaborate on the reasons for doing so. The team agreed that these videos provided interesting insight into the phenomenon of concealment, although they did not clearly answer the research question (what are the motivations for concealment?). To balance the clinically relevant information obtained from the thematic analysis of these videos while answering our research question, the theme “Feelings about Disclosure” is discussed as an exploratory result. The 8 videos that did not discuss motivations are removed from calculations in Table 2.

**Table 2.** Themes and subthemes constructed for videos about the concealment of psychiatric symptoms and their frequency. This table represents the 90 videos that discussed reasons for concealment. The number of videos reported in each theme represents the number of unique videos per theme (ie, videos with more than one subtheme were not double-counted).

Themes and subthemes	Videos, n (%)
Disclosure as punitive	31 (34.4)
Hospitalization after honest disclosure	9 (10)
Fear of hospitalization	4 (4.4)
Desire to avoid hospitalization	17 (18.8)
Desire to leave inpatient hospitalization	2 (2.2)
Managing others’ feelings and impressions	28 (31.1)
Fear of upsetting therapist	5 (5.5)
Fear of upsetting/disappointing parents	3 (3.3)
Parents knowing about symptoms	8 (8.8)
Maintaining façade	7 (7.7)
False therapeutic improvement leading to perceived progress	9 (10)
Feeling bad and feeling nothing	21 (23.3)
Feeling alone/silent suffering	7 (7.7)
Perceived burdensomeness	4 (4.4)
Hopelessness	2 (2.2)
Patient fear that they are faking illness	3 (3.3)
Fear of being vulnerable in therapy	6 (6.6)
Minimizing symptoms to self	2 (2.2)
Inability to access emotions	2 (2.2)
Negative opinions of psychiatric treatment	17 (18.8)
Perceived lack of patient-therapist confidentiality	3 (3.3)
Treatment does not work	2 (2.2)
Hospital as restrictive	3 (3.3)
Issues with psychiatric treatment structure and modality	8 (8.8)
Weak therapeutic alliance	7 (7.7)

## Results

### Overview

**Figure 1.**
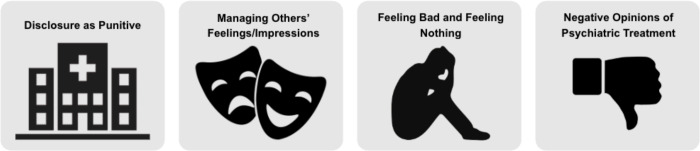
Key themes identified in TikTok videos about concealing psychiatric symptoms.

Four predominant themes regarding reasons for concealing psychiatric symptoms were created: (1) Disclosure as Punitive, (2) Managing Others’ Feelings and Impressions, (3) Feeling Bad and Feeling Nothing, and (4) Negative Opinions of Psychiatric Treatment. Each theme had multiple subthemes that often overlapped, as described below, and most videos contained more than one theme. Table 2 shows the themes, subthemes, and percentages of videos with each theme and subtheme, and Figure 1 visualizes the themes. Table 3 summarizes the distribution of the number of codes assigned per video.

**Table 3.** Distribution of codes assigned to each TikTok video in the sample. This table calculates code assignments for the entire sample of 98 videos.

Number of codes assigned per video	Videos, n (%)
1	41 (42.3)
2	37 (38.1)
3	9 (9.3)
4	1 (1.0)
5	1 (1.0)

### Statistical Analysis

At the time of download, the 98 videos that comprised our sample had a combined 73,252,531 views, 14,356,874 likes, 74,954 comments, 770,027 shares, and 1,204,006 saves. Numbers were rounded to the nearest tenth of a thousand when recorded (eg, 13.5 k) to reflect how TikTok displays video metrics. Table 4 provides the average, SD, median, and range across videos. Each video was posted by a unique creator.

**Table 4.** Mean, SD, median, and range of TikTok videos about concealment of psychiatric symptoms. These metrics evaluate the entire sample of 98 videos.

Metric	Mean (SD)	Median (range)
Views	755,181 (1,596,059)	94,100 (90‐7,400,000)
Likes	159,521 (339,147)	10,950 (9‐1,700,000)
Comments	781 (1773)	64 (0‐9282)
Shares	8021 (29,168)	188 (1‐225,100)
Saves	13,682 (31,094)	1554 (1‐198,100)

### Theme 1: Disclosure as Punitive

The majority of videos in our sample reflected an attitude that disclosure has negative consequences for the creator, specifically being hospitalized. Most videos communicated a desire to avoid experiencing an involuntary inpatient hospitalization, with 4 creators explicitly mentioning that concealment of symptoms or the severity of symptoms was due to fear of being hospitalized. Most of these videos followed a similar structure, including the sentiment that the creator lies to their therapist so that they can avoid the hospital. Nine videos recounted being hospitalized as a result of honest disclosure. One video showed a creator in a hospital gown slamming a pillow on the floor with audio of someone crying and screaming. Text on the screen read, “POV: You told your therapist your real thoughts.” Other videos showed scenes from a hospital with captions like “I was too honest with my therapist.” Two videos talked about symptom concealment as a means to leave inpatient hospitalization. The sentiment that the hospital is a restrictive place (eg, “grippy sock jail”) also seemed to shape disclosure considerations.

### Theme 2: Managing Others’ Feelings and Impressions

Videos with this theme discussed how other people factored into their decision-making about disclosure. Eleven videos specifically talked about parents and family. Some creators considered other people’s emotional responses to their symptom disclosure when deciding whether to report or conceal their symptoms. Five creators talked about being afraid their therapist would be upset by or unable to handle their emotions. One video is a cartoon depicting a creator’s experience disclosing to their therapist who “cri[ed] after hearing [their] story for the first time.” As a result, the creator says that they felt “guilty immediately” and felt “bad for making them cry and stop[ped] talking completely.” Three creators mentioned wanting to shield their parents from disappointment or distress that could result from their disclosure of symptoms. One said, “My mom sent me to therapy & i lied to the therapist the entire time so she didn’t see me as a disappointment.”

Creators also articulated efforts to curate an image of themselves as “well.” One such effort included lying about symptoms in therapy so that the therapist would think that they were better, which often led to premature treatment termination or a reduction in treatment frequency. One creator said, “I love when I have therapy and I lie to my therapist and they’re like ‘oh you seem to be doing really great’ or ‘making good progress.’ I’m like yeah. That’s because of the lies that you think that.” The creator captioned this video, “was hoping you would see right through me.” Other efforts to appear well included endeavoring to make their life seem fun or putting extra effort into how they dress. Not all creators explicitly discussed their motivations for making themselves out to be less symptomatic than they were, but simply discussed doing so. Creators also spoke about not wanting their parents to know that they were experiencing psychiatric symptoms, and often not providing a rationale. For example, creators mentioned lying about their symptoms because their moms were in the room. Finally, 7 videos included a discussion of projecting and maintaining a façade of wellness to the outside world that conflicted with their internal emotional state. One creator responds to the statement “we didn’t know you were struggling that much.” by lip syncing along to lyrics saying, “I made it all look painless/Man am I the greatest.”

### Theme 3: Feeling Bad and Feeling Nothing

When talking about disclosure, creators mentioned experiencing their negative emotions in silence, and feeling alone, unsupported, burdensome, and hopeless. For example, when discussing disclosure to a therapist, one creator said, “Who really admits the truth when all we’ve know [*sic*] is to fight on our own.” Creators also shared efforts to suppress these negative emotions. They expressed fear of being vulnerable in therapy and an impulse to minimize their symptoms within their self-talk. One creator filmed herself next to text reading, “Me staying silent in therapy so I don’t start crying.” In other videos, creators shared that they experience an inability to access and identify emotions. Two creators explained that they do not disclose because they do not know what they are feeling. In one video, the creator lip-synced to audio that said, “I’m going to kill myself” alongside text that read, “when i couldn’t tell my therapist what was wrong because i didn’t even know what was wrong.” One other code was patients expressing fear that they were faking their psychiatric illnesses, even when they did not believe this to be the case. In one example of such a video, a creator recorded herself laughing alongside text that says, “*when you think you’re faking your mental illness but then doctors tell you its serious.*”

### Theme 4: Negative Opinions of Psychiatric Treatment

Most videos in this theme included discussion about creators’ issues with normative aspects of how psychiatric treatment is structured. One creator mentioned being suspicious of treatment due to its cost, endorsing the notion that therapists only care because they are paid. Another creator mentioned disliking that psychiatrists focus on medication management, while another took issue with the fact that therapy is only once a week. Yet another was frustrated with the psychotherapeutic practice of Socratic questioning. For example, one creator filmed herself praying alongside audio repeating “Don’t say it” on a loop with text on the video reading, “Every therapist after we tell them a *story*.” The video ended with the text, “And how did that make you FEEL?” Creators also worried about a lack of patient-therapist confidentiality, sometimes due to the ethical mandate that confidentiality will be broken if a patient is an imminent risk to themselves. Combining the subthemes that treatment does not work and therapy lacks true confidentiality, one creator posted a video of herself smiling with the text, “me leaving therapy knowing all i did was lie bc even therapist [*sic*] tell other ppl and telling someone how i feel does nothing.” Seven videos mentioned a weak therapeutic alliance as a reason for nondisclosure.

### Exploratory Thematic Analysis: Feelings About Disclosure

While the purpose of this study was to examine reasons for concealment of psychiatric symptoms, some creators talked about their feelings about the concealment of psychiatric symptoms. Six creators mentioned being honest in therapy, expressing confusion as to why someone would lie to their therapist. Five videos depicted creators being satisfied about not disclosing, often smirking at the camera, and joking about tricking their therapist. Creators experienced negative feelings due to not disclosing, including guilt due to lying to their therapist and hopelessness about not disclosing. One creator filmed herself looking sad alongside text that said, “That feeling of guilt after blatantly lying to your therapist <<” with the caption, “Some things I’ll never be able to say.” Another video showed a girl’s face morphing from a neutral expression to one with tears and mascara running down her cheeks. The neutral face had the text “Me after always lying to my therapist so she thinks I’m getting better,” while the crying face had text reading, “Knowing now that I’ll never get my true feelings out.” In this video, she is showing the façade she is presenting to her therapist and the resulting hopelessness about getting better. Other creators struggled to identify why they could not be honest with their provider, but confirmed that they were being dishonest. Three creators talked about being ambivalent about whether to disclose. In one video, a creator filmed themselves filling out a self-report questionnaire of psychiatric symptoms and moving their pencil back and forth between “not at all” and “very often” before answering every question with “not at all.”

## Discussion

### Principal Findings

This study sought to understand why patients withhold reporting or minimize psychiatric symptoms in clinical encounters using user-initiated content from TikTok. Through reflexive thematic analysis of 98 TikTok videos, we identified 4 overarching themes describing reasons for withholding or minimizing psychiatric symptoms in clinical encounters: Disclosure as Punitive, Managing Others’ Feelings and Impressions, Feeling Bad and Feeling Nothing, and Negative Opinions of Psychiatric Treatment. These themes and their subthemes show that patients have diverse and multifaceted motivations for concealing psychiatric symptoms. The most frequently endorsed reasons for concealment were specific perceived features of psychiatric treatment, including fear of involuntary hospitalization and desire to avoid the hospital. Interpersonal concerns, such as not wanting to upset family members or therapists, or wanting to present as “doing well,” were also barriers to disclosure. Some creators struggled to identify and express their feelings, while others endorsed shame, hopelessness, or a fear that their symptoms were not “real enough” to merit care. These findings extend prior research by examining concealment outside laboratory or interview contexts and provide novel insight into decision-making about concealment and disclosure.

### Disclosure as Punitive

The most prevalent theme was Disclosure as Punitive. Results indicated that negative perceptions of treatment, such as seeing the hospital as restrictive and issues with psychiatric treatment structure and modality, may relate to desires to minimize symptoms. The most prominent reason for lack of disclosure expressed in our sample was the desire to avoid hospitalization. This observation parallels previous work suggesting that evading psychiatric hospitalization is a key barrier to disclosure of STBs [4,5,13,15,17]. Prior work suggests that one reason for the desire to avoid hospitalization in people admitted for STBs is perceived lack of utility and negative experiences [47]. These sentiments have been echoed in qualitative work describing TikTok content about treatment for eating disorders [24]. Low satisfaction with inpatient stay has been associated with nondisclosure of STBs in quantitative work as well [11]. Creators also talked about being hospitalized after honest disclosure, often suggesting that they regretted being honest with their clinician due to this outcome. Indeed, some patients perceived hospitalization as a punishment for honest disclosure. To align patient and clinician understanding, it may be helpful for clinicians to address what “imminent risk” looks like for each patient. Since STBs are idiosyncratic [48], having a predetermined agreement about when hospitalization would be necessary before a suicidal crisis is warranted. In one example of doing so, clinicians who experienced nondisclosure with their military-affiliated patients dealt with this challenge by normalizing the occurrence of STBs and collaborating on limiting means [49]. Additionally, clinicians should discuss and uncover what patients’ concerns about hospitalization are to help correct misinformation and reduce anxiety about hospitalization. Patient fears may be addressable or unfounded in what occurs on an inpatient unit. These findings suggest that suicide risk management practices perceived as coercive may unintentionally create incentives for concealment, potentially compromising accurate detection of suicide risk.

### Managing Others’ Feelings and Impressions

Creators voiced desires to manage other people’s feelings and impressions. Creators described feeling that their emotions were too intense for others to handle and would provoke distress, which in turn elicited guilt. Not wanting to worry others as a reason for nondisclosure has been reported previously [13,50], and prior work has shown modest effects of perceived burdensomeness in predicting suicide-related outcomes [51,52], suggesting shared risk factors for nondisclosure and suicide. Relationship dynamics may also shape disclosure and explain why many creators discussed wanting to conceal information from parents. For example, families’ reduced ability to respond to one another’s emotional needs with appropriate affect has been linked to negative psychiatric outcomes [53,54]. Future research should examine how family responses to emotional expression influence patterns of disclosure—whether nondisclosure emerges as a learned behavior in emotionally suppressive families or from experiences of others reacting intensely to one’s needs. It may also be informative to investigate whether targeting perceived responsibility for managing others’ emotions in treatment could reduce nondisclosure.

Creators also communicated endeavors to be seen by others as psychologically well. These efforts did not seem to be a deception to avoid disclosure but rather a form of impression management. Work in nonclinical populations suggests that people in general are motivated to shape the way they are perceived by others through impression management, a process wherein people deliberately control how others perceive them to achieve social, emotional, or identity-related goals [21,55,56]. Aligning with our observation that people are motivated to be perceived as healthy, Leary and Kowalski [56] maintain that people are motivated to engage in impression management to obtain optimal social outcomes, such as acceptance from others, increased subjective well-being and self-esteem, and a strong identity. This may extend to social outcomes, including not being hospitalized.

Indeed, work on impression management has been extended to clinical populations; patients engage in impression management during intake interviews [57]. Because psychopathology has long been stigmatized in our society [58], these efforts at impression management are likely adaptive because stigma, including self-stigma, is related to negative health outcomes [59]. Particularly, STBs are quite stigmatized [60,61]. Such stigma yields a reciprocal relationship between experiences of suicide stigma and increased suicidality [60,62,63]. Therefore, avoiding being seen as suicidal may have adaptive consequences, particularly if a patient has previously observed or experienced negative socioemotional consequences of suicide stigma [64]. However, when stigma prevents people from seeking treatment, it in turn engenders a greater risk of suicide. One study did, in fact, identify stigma as a barrier to disclosure [49]. Taken together, our observation that impression management impacts disclosure may reflect a common human tendency to minimize expressions of distress to avoid unwanted social outcomes, likely due at least in part to the stigma of mental illness and ego-dystonic self-perceptions. Clinicians should discuss this tendency and perceptions of mental health stigma with their patients. This may help address stigma directly and highlight the harms of impression management and the importance of disclosure.

These findings indicate that disclosure decisions are shaped not only by individual symptom severity but also by relational dynamics and anticipated emotional consequences, underscoring the importance of explicitly addressing perceived burdensomeness, stigma, and impression management within therapeutic relationships.

### Feeling Bad and Feeling Nothing

Creators reported that negative affect impacted their desire to disclose mental health symptoms. Loneliness, burdensomeness, and hopelessness are well-documented risk factors in the suicide literature [65,66]. Similarly, creators’ discussion of emotional suppression aligns with literature suggesting that STBs are motivated and maintained by an intolerance of negative affect; people with STBs and nonsuicidal self-injury (NSSI) endorse a desire to avoid and escape negative affect [67-69]. This suggests common risk factors between STBs and nondisclosure, which have been found previously. Help-negation, a phenomenon where people with high levels of SI decrease their help-seeking behavior, has been observed in young people with SI [70,71]. This behavior is a key impediment to suicide risk detection and treatment. Our work provides needed insight into the underpinnings of the phenomenon, which was not simply explained by hopelessness or prior help-seeking in previous work [70].

While this study did not find shame as a subtheme, a common self-reported reason for concealment in therapy also includes the desire to avoid shame [5], similarly suggesting a lack of tolerance for negative affect, factoring into the decision-making process about disclosure. It may be that people who feel a lot of shame surrounding disclosure are unlikely to post about their emotions online. Indeed, there is an interesting dissonance between the finding that people often do not disclose to their clinicians while actively disclosing to peers and a larger and unknown internet audience. It may be that disclosing to those without the power to hospitalize or peers is less difficult. It also may be that the subset of individuals who withhold information from their therapist yet disclose similar content to large online audiences is not representative of the broader population of individuals who conceal information in therapeutic contexts. Finally, creators expressed not knowing how they felt as an explanation for why they did not share information, aligning with literature suggesting that alexithymia is related to depression and NSSI [72,73]. Taken together, patients’ symptoms themselves may be a barrier to disclosure, even if they have a desire to confide in their clinician.

One unexpected subtheme was patients’ fear that they were faking their mental illness. Some seemed to be afraid of this possibility despite feeling ill, while others seemed to be in denial of their symptom severity until a clinician highlighted it. Another group of patients may be using self-invalidation as an attempt at denial via minimizing their symptoms to themselves. Previous work suggests that there is both self-stigma and perceived stigma surrounding perceived depression inauthenticity among adolescents online, which presented as a barrier to help-seeking [74]—this phenomenon may extend to STBs. Creators in our sample who feared that they were faking their symptoms may have been experiencing such self-stigma. It is also possible that creators are expressing a version of the thought that their SI is not that serious, which has been reported elsewhere as a reason for nondisclosure of STBs [13,50]. Of note, obsessive fears that one is unintentionally a liar, such as fear that one is “faking” a disorder, are seen in patients with obsessive-compulsive disorder [75]. More work is needed to understand this phenomenon. Psychoeducation about the importance of disclosing STBs in therapy, no matter the severity, may be warranted.

### Negative Opinions of Psychiatric Treatment

Creators also discussed barriers to disclosure that related to the therapeutic relationship. Because a weak therapeutic alliance was a theme related to disclosure, building rapport is likely to be critical to build trust and reduce negative perceptions of treatment to create an environment where patients feel comfortable disclosing. Previous work suggests that a strong therapeutic alliance increases the likelihood of honest disclosure, and therapists’ emotional responses to patients were correlates of disclosure [5,11,15]. Clinicians may benefit from providing extensive psychoeducation about the therapeutic relationship and the rationale for norms of psychiatric care, as well as inviting patients to share reservations they have about psychiatric treatment in order to have an open discussion about them. Additionally, increased transparency from clinicians may be helpful. For example, clinicians should clearly state when they will or will not be giving advice and why they are choosing to see a patient at a certain frequency. Previous work suggests that one reason underlying the lack of disclosure is a feeling that their clinician would not understand a particular issue [5], suggesting that building the strongest possible foundation of trust and understanding in the therapeutic relationship is the most crucial for this population, as the outcome is a matter of life and death. Notably, some creators also reported therapist misconduct, such as an instance where a therapist cried after a creator’s disclosure. This finding echoes previous work that suggests people conceal information to avoid therapists’ potential overreactions [5]. Considering this finding, clinical training should continue to emphasize appropriate and effective ways of building rapport and managing countertransference. Future work should also examine whether certain clinician characteristics predict patient nondisclosure or minimization. Additionally, patient dishonesty was associated with weaker therapeutic alliances and worse perceived therapeutic outcomes [76], suggesting that psychoeducation about the importance of disclosure may be warranted.

### Feelings About Disclosure

In our exploratory thematic analysis, we found that a paucity of creators talked about being honest with clinicians. While this is likely due to our search terms, it is also possible that people are engaging in self-presentation to fit in with a sentiment that lying to clinicians is normative. Indeed, work shows that social media, including TikTok, may positively reinforce discussion of mental health symptoms by allowing users to gain social capital, attention, and belonging [77-79]. However, these symptoms may be performative or exaggerated for positive gain when identifying with psychopathology is perceived as desirable in online spaces [79]. While it is possible that discourse about nondisclosure has a similar function to identification with mental health symptoms, it seems more likely that our findings are best explained by the specificity of our search terms. As observed during our exploration of potential search terms, there is no unifying hashtag or term that discourse about nondisclosure to clinicians centers around. Thus, it is unlikely that sharing these narratives online is motivated by wanting to fit into an online social group shaped by a popular “sick role” narrative. Furthermore, creators expressed negative emotions as a result of nondisclosure. This suggests that clinicians may benefit from candid conversations with patients about the potential long-term effects of keeping symptoms private.

### Broader Implications

Taken together, findings suggest that concealment may be an attempt to protect oneself from feared or actual negative consequences grounded in patients’ previous treatment experiences or in cultural narratives about psychiatric care. Further work is needed to understand the downstream effects of expressing negative treatment experiences online. It may be the case that learning about others’ disclosure of STBs leading to hospitalization on TikTok can influence one’s willingness to disclose their STBs to their therapist. Future work should investigate this possibility. It is currently unknown whether this content discourages help-seeking and entrenches negative perceptions of treatment—both on the part of the creator and the viewer—or provides validation that heightens feelings of connection.

Future work should examine what differentiates people who do disclose from those who view disclosure as dangerous or futile, perhaps through protective traits, skills, or relational contexts. Prior work suggests that disclosure patterns vary across settings and individual characteristics. In one study, neuroticism and trait anxiety predicted disclosure to research assistants, while lower extraversion predicted disclosure to clinicians. Extraversion and trait anxiety distinguished disclosure to research assistants from disclosure to clinicians [20]. Other studies highlight individualized psychological barriers to disclosure. People with self-destructive behaviors, defined as self-harm, substance abuse, or disordered eating, reported greater self-reported difficulty being honest in therapy compared with peers without such behaviors who also reported difficulty being honest in therapy [80]. Nondisclosure was also associated with social loneliness, poor health, frequent SI, and severe psychological distress [81], as well as personality disorder diagnoses and symptoms of suicide crisis syndrome [11]. On the other hand, reasons for disclosure have included help-seeking, shared background, catharsis [13], a strong therapeutic alliance, and intentions to address STBs [15]. Building on these findings, interventions such as motivational interviewing may help leverage motivations to promote disclosure.

### Limitations and Future Directions

This study highlights several paths for future research. Work should examine whether improving emotional awareness and tolerance of negative affect increases disclosure, or whether people simply disclose more as symptoms improve. Given links between alexithymia, depression, and NSSI, interventions that enhance emotional articulation may facilitate disclosure and rapport. Future studies should also explore fears of “faking” illness as potential expressions of self-stigma, anxiety, or intrusive thoughts, and how family dynamics shape beliefs about managing others’ emotions in clinical settings. Studies should also examine how online narratives about psychiatric care influence help-seeking and identify what differentiates patients who disclose honestly in clinical settings from those who do not. Finally, using standardized measures for assessment may also help surmount barriers to disclosure [49]. Particularly, rather than force a response, clinicians may allow for a nondisclosure response (eg, prefer not to answer) option in both self-report measures and during sessions [82]. To enhance the rigor of qualitative social media research, the field may benefit from developing structured scoring procedures to assess short-form video quality for qualitative analyses, comparable to the Global Quality Score or DISCERN [83].

Because TikTok users tend to be younger, some themes we uncovered may be adolescent or young adult specific. Indeed, adolescents and young adults may have unique reasons for choosing not to disclose, including fear of negative reactions from family and a sense of self-reliance [18]. In addition, there may be selection bias based on who decides to post online. People who feel intense shame and stigma, are more introverted, or have higher levels of social anxiety may not choose to share their experiences online. Indeed, a bidirectional relationship between shame and nondisclosure has been shown in a nonclinical sample [84]. This study also lacks information on how many clinical encounters discussed in our TikTok videos were in-person versus telehealth. Telehealth may prohibit clinicians from observing evidence of self-injurious behaviors on patients’ bodies. As such, clinicians may be missing key clues that could initiate a conversation. Additionally, people may struggle to conceal in person but have an easier time doing so online. Future work should address this possibility. Another limitation of this study is that our data scraping procedures did not capture the dates when videos were posted, preventing us from examining temporal trends or shifts in public discourse over time. Although reporting dates are not common in work examining TikTok, future work may benefit from looking at the same search terms longitudinally to explore trends. Further, demographic information was not systematically available due to video filters, green screen, and other effects, and video formats. As a result, we were unable to examine whether motivations for concealment differed by demographic characteristics such as race, ethnicity, and gender identity.

Finally, the biggest limitation of this study was that the specific symptoms that patients were concealing were sometimes unknown. TikTok censors terms such as suicide and self-harm, and creators in our sample did not always explicitly state their symptoms. When creators were not explicit, we were often able to ascertain the creators’ symptoms anyway. Creators often alluded to STBs (eg, the creator mouthed the word “suicide,” gestured to slitting their throat, or included an oblique hashtag such as #sewersl1de). In addition, in videos that discussed adult involuntary hospitalization, the coding team conjectured that symptoms were related to being a danger to self or others. Future work should replicate this work on a less censored platform. Finally, given the observational nature of this work, we cannot verify the veracity of creator narratives, including whether they visited a health care provider or are in fact experiencing the symptoms they discuss concealing.

### Conclusions

Thematic analysis of naturalistic, user-generated social media content suggests that concealment of psychiatric symptoms is often motivated by fear of punitive consequences, stigma, interpersonal dynamics (within family, treatment team, and one’s broader social context), and dissatisfaction with treatment practices. These findings highlight modifiable relational and structural factors that may influence disclosure in clinical contexts. Efforts to improve transparency around hospitalization criteria, strengthen the therapeutic alliance, and directly address fears of stigma may reduce concealment and enhance accurate detection of psychiatric symptoms. This is critical to reduce death by suicide in people who voluntarily present to our hospital systems. Future work should examine how institutional policies and clinician communication strategies shape patients’ willingness to disclose suicide risk.

## Abbreviations

NSSI: nonsuicidal self-injury
SI: suicidal ideation
STB: suicidal thoughts and behavior
